# Case Report: Growth hormone deficiency and response to treatment in MIRAGE syndrome: expanding the endocrine phenotype

**DOI:** 10.3389/fendo.2026.1885153

**Published:** 2026-07-07

**Authors:** Laura Trapani, Marta Cognigni, Natalia Maximova, Erica Valencic, Alice Fachin, Gianluca Tamaro, Alberto Tommasini, Gianluca Tornese

**Affiliations:** 1 Department of Medical Sciences, University of Trieste, Trieste, Italy; 2 Institute for Maternal and Child Health IRCCS “Burlo Garofolo”, Trieste, Italy

**Keywords:** adrenal insufficiency, case report, growth hormone, growth restriction, myelodysplasia, SAMD9, MIRAGE, somatropin

## Abstract

**Background:**

MIRAGE syndrome, a rare autosomal dominant disorder, is caused by heterozygous gain-of-function mutations in the SAMD9 gene. A key characteristic of MIRAGE syndrome is growth restriction. Although initially thought to stem mainly from prenatal and systemic factors, this growth restriction can also be a consequence of panhypopituitarism, leading to growth hormone deficiency (GHD). The use of recombinant human growth hormone (rhGH) to treat the characteristic severe growth failure is controversial due to an inherent risk of myelodysplastic syndrome (MDS) and acute myeloid leukemia.

**Case presentation:**

We report an 11-year-old male diagnosed with MIRAGE syndrome confirmed by a heterozygous *de novo* SAMD9 variant (c.4615T>A; p.Leu1539Ile) who had previously undergone allogeneic hematopoietic stem cell transplantation for MDS with monosomy 7. Severe pre- and postnatal growth restriction, characterized by short stature and slow growth velocity, marked the clinical course. Comprehensive hormonal testing was performed and ultimately revealed a growth hormone deficiency (GHD). At age 6, growth hormone therapy began after a brain MRI to assess the pituitary gland anatomy. This decision followed a comprehensive risk-benefit analysis and a hematological evaluation that showed no signs of clonal evolution. Over a six-year follow-up period, the patient demonstrated a significant improvement in growth velocity and height standard deviation score, with stable hematological parameters and no adverse events.

**Conclusion:**

This case expands the known endocrine phenotype of MIRAGE syndrome, providing the first report, to our knowledge, of a favorable and safe medium-term response to rhGH therapy in this condition. Our observations support systematic GH stimulation testing in MIRAGE patients with marked growth failure who survive beyond early childhood and suggest that, in carefully selected cases with proven GHD, rhGH replacement may be considered in close collaboration with hematology/oncology teams and under strict hematological monitoring.

## Introduction

MIRAGE syndrome (OMIM: 617053) is a severe and exceptionally rare congenital disorder, first described as a distinct clinical entity in 2016, with an estimated prevalence of less than 1 per 1,000,000 live births (1). The syndrome’s name is an acronym that encapsulates its six cardinal features: Myelodysplasia (M), recurrent Infection (I), Restriction of growth (R), Adrenal hypoplasia (A), atypical Genital phenotypes (G), and Enteropathy (E) (1–3).

The clinical picture is usually dramatic, starting in the neonatal period. Infants are often premature and suffer from significant intrauterine growth restriction. They demonstrate persistent postnatal growth failure, with weight, length/height, and head circumference typically staying below –2.0 SDS, even with sufficient caloric intake (4).

Early childhood mortality is high, most commonly due to invasive infection (5). The subsequent clinical course is often complicated by severe, life-threatening events, including adrenal crises, profound failure to thrive, and severe infections resulting from defects in both hematological and innate immunity (3).

The genetic basis of MIRAGE syndrome is attributed to heterozygous germline gain-of-function (GoF) missense mutations in the Sterile Alpha Motif Domain-containing 9 (*SAMD9*) gene, located on chromosome 7q21.2 (5). These mutations almost always arise *de novo*, although rare instances of inheritance from an asymptomatic parent with somatic mosaicism have been documented (4).

The SAMD9 protein is a ubiquitously expressed cytoplasmic protein that acts as a potent endogenous growth repressor by restricting cell proliferation and protein translation, and it also functions as an interferon-inducible innate effector that contributes to antiviral defense, in part through the formation of cytoplasmic antiviral granules and sensing cytosolic nucleic acids (6).

The GoF mutations identified in MIRAGE syndrome do not inactivate the protein but rather pathologically enhance its intrinsic antiproliferative activity (2). This hyper-repressive function is now regarded as the central driver of the disease’s pathophysiology, leading to the widespread tissue and organ hypoplasia that manifests clinically as growth failure, adrenal hypoplasia, and other developmental defects (1).

A critical and seemingly paradoxical aspect of MIRAGE syndrome is the markedly increased risk of myeloid malignancies, particularly myelodysplastic syndrome (MDS) and acute myeloid leukemia (AML), despite being caused by a GoF mutation in a tumor suppressor gene. This occurs through a powerful process of *in vivo* negative selection and somatic evolution. The severe growth restriction imposed by the mutant SAMD9 generates strong selective pressure, particularly in rapidly dividing hematopoietic cells, to acquire secondary lesions that alleviate this antiproliferative effect (7–9).

The most commonly observed escape mechanism is the loss of the entire chromosome 7 that carries the mutant *SAMD9* allele, a phenomenon termed “adaptation by aneuploidy”. The resulting state of monosomy 7 is one of the most well-established high-risk cytogenetic abnormalities for the development of MDS and subsequent leukemic transformation (9). Therefore, the biological mechanism that permits hematological survival concurrently establishes a pre-leukemic state, representing a profound and clinically crucial biological trade-off (1).

Although primary adrenal insufficiency was considered a core manifestation of MIRAGE syndrome, a substantial subset of patients, in some cohorts around 20-30%, do not present with primary adrenal insufficiency (PAI), a feature once considered nearly universal (1, 4, 10). These individuals without an adrenal phenotype tend to have a higher birth weight and are born at a later gestational age, suggesting a potentially milder disease course (3).

The spectrum of associated clinical features continues to expand and now includes renal dysfunction (e.g., nephrotic syndrome, focal segmental glomerulosclerosis), neurological complications (e.g., global developmental delay, hydrocephalus, perivascular calcifications), gastroenterological manifestations (e.g., achalasia, gastroesophageal reflux, recurrent intussusception), ophthalmological findings (e.g., hypolacrimia and corneal anesthesia) and autonomic dysfunction (11–19). Furthermore, the spectrum of endocrinological manifestations is now broader. In addition to adrenal hypoplasia and gonadal dysgenesis, reported cases now encompass, among others, hypothyroidism, hypo- and hyperglycemia, primary hypoparathyroidism, and panhypopituitarism (4). Within this expanding endocrine spectrum, the potential contribution of pituitary dysfunction and GHD to growth failure in MIRAGE remains poorly characterized, with virtually no data on rhGH replacement.

The primary objective of this report is to provide detailed clinical, biochemical, and genetic characterization of a patient with MIRAGE syndrome who presented with severe growth failure. To date, data on growth hormone therapy in MIRAGE syndrome are extremely limited, and no clear association with increased leukemic transformation has been demonstrated, although theoretical concerns remain (10). In this patient, growth hormone deficiency (GHD) was documented, and treatment with recombinant human growth hormone (rhGH) was initiated. We discuss the safety and efficacy of rhGH therapy in this case, despite the significant therapeutic dilemma associated with managing this condition, due to the theoretical risk of promoting malignant transformation in individuals with an underlying predisposition to MDS. To our knowledge, this is the first report of rhGH therapy in a genetically confirmed MIRAGE patient with documented GHD.

## Case presentation

The patient is a 46, XY male child born to healthy, non-consanguineous parents. The pregnancy was complicated by marked fetal growth restriction (*R*, Restriction of growth) identified on a routine anomaly scan at 22 weeks of gestation. The child was born prematurely at 33 + 1 gestational weeks via emergency caesarian section for fetal distress represented by late decelerations. His birth weight was 1,490 g (-1.22 SDS), length was 42.5 cm (-0.4 SDS), head circumference 28.5 cm (-1.4 SDS). The neonatal course was complex and characterized by respiratory distress syndrome and neonatal sepsis; therefore, he was admitted to the Neonatal Intensive Care Unit (NICU). During the hospitalization in the NICU, which lasted 2 months, thrombocytopenia and anemia were additionally observed.

From the first months of life, he exhibited recurrent upper respiratory infections, gastroenteritis, otitis media, and sepsis (*I*, recurrent Infection), leading to multiple hospitalizations. He was also suffering from cramping abdominal pain, watery diarrhea, requiring intravenous hydration, and intussusception, requiring surgery at the age of 5 months (*E*, Enteropathy). Due to recurrent infections, a bone marrow aspirate and biopsy were performed at 2 years of age, revealing myelodysplastic syndrome (*M*, Myelodysplasia) with monosomy of chromosome 7 and combined immune deficiency.

Immunophenotype showed abnormal lymphocyte subsets, with near absent peripheral B cells (0.15% of lymphocytes, reference range 17,3%-30,3%, almost all with a memory phenotype), low percentages of natural killer cells (0.5%, reference range 4%-17%), low CD4/CD8 ratio (0.38, reference range 1.3-3), low recent thymic emigrants (CD45RA+CD31+ cells = 22.2% of CD4+ cells, reference range 52.3%-66.6%). Expression of activation markers (CD25+ and CD69+) after *in vitro* stimulation with mitogen or anti-CD3+/CD28+ antibodies was normal.

Therefore, an allogeneic hematopoietic stem cell transplantation (HSCT) from an HLA-matched AB0-mismatched unrelated donor was successfully performed at the age of 2 years and 3 months after treosulfan/fludarabine/thiotepa/antithymocyte globulin (ATG) conditioning. On day +28, full donor chimerism was documented in the peripheral blood. He developed chronic lung disease characterized by bronchiectasis and multiple parenchymal infiltrations. He also showed mild global developmental delay and was diagnosed with periventricular leukomalacia and autism spectrum disorder. When he was almost 6 years old, whole-exome sequencing was performed on DNA extracted from a skin biopsy to circumvent donor hematopoietic chimerism, revealing a *de novo* variant (c.4615T>A, p.Leu1539Ile) in the *SAMD9* gene – previously described in the literature (8) – with a unifying diagnosis of MIRAGE syndrome. Retrospectively, these multisystem manifestations fulfilled all the cardinal MIRAGE features (Myelodysplasia, recurrent Infection, Restriction of growth, Adrenal hypoplasia, atypical Genital phenotypes, and Enteropathy).

**Figure 1:**
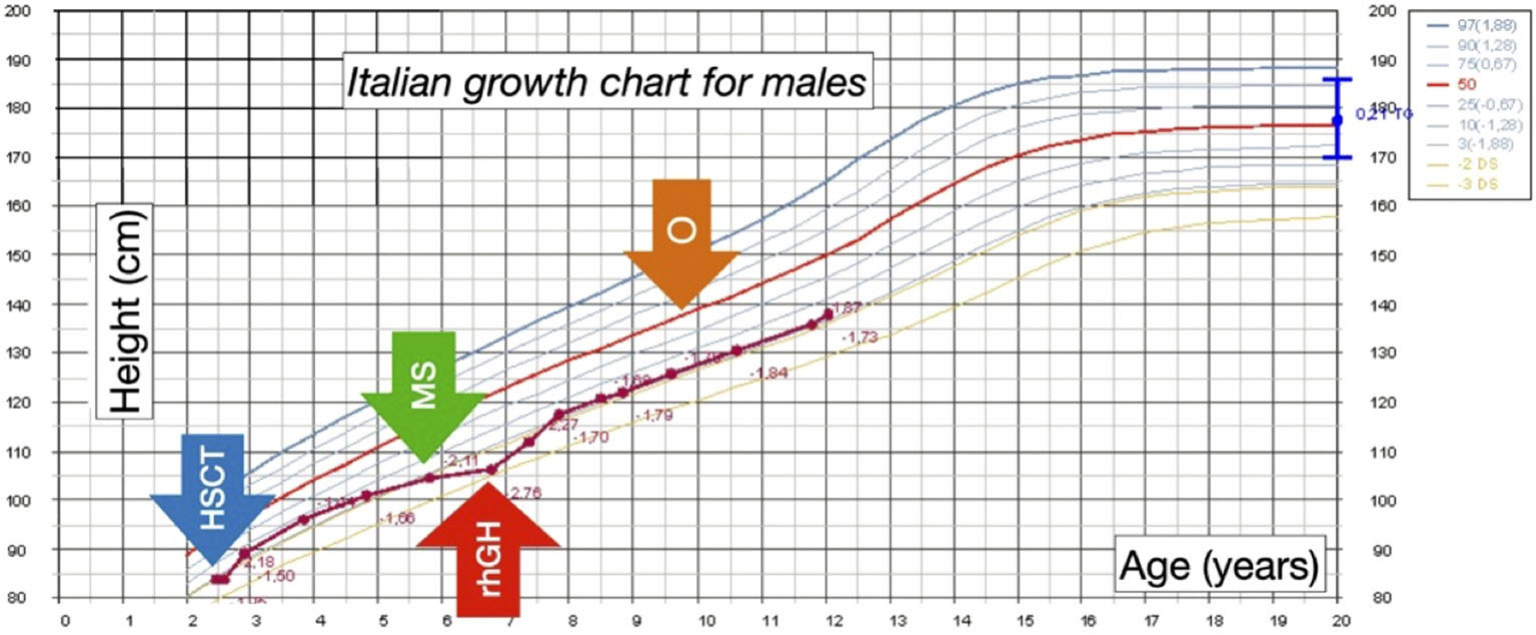
Growth chart of the patient plotted on the Italian growth reference for males (years on the X-axis, height in cm on the Y-axis). Arrows indicate significant events and the age at which they occurred: hematopoietic stem cell transplantation (HSCT), MIRAGE syndrome (MS) diagnosis, initiation of recombinant human growth hormone (rhGH) therapy, and orchidopexy (O). The dark red line with circles represents the patient’s height measured at each visit; the values next to the circles indicate height expressed as Standard Deviation Score (SDS) for age and sex. The mid-parental height is shown as a blue horizontal bar, with the central dot indicating the calculated target height (177.8 cm, −0.21 SDS).

He was referred to pediatric endocrinology at the age of 6 years and 8 months to assess the presence of adrenal hypoplasia, genital phenotype and to investigate the growth restriction, considering the presence of short stature (-2.76 standard deviation scores [SDS]) and slow growth velocity over the previous two years (1.76 cm/year, -4.56 SDS) *(R)* (**Figure 1**) and normal weight (BMI 16.14 kg/m^2^, -0.06 SDS). Tanner’s pubertal stage was G1, Ph1 with retractile small testes (volume <1 ml) (*G*, atypical Genital phenotypes) and normal male external genitalia.

To assess the presence of adrenal insufficiency, several blood tests and an abdominal MRI were performed. The magnetic resonance imaging showed adrenal glands within size limits for height and weight. Basal ACTH and cortisol levels were measured and found to be within the normal range (10.6 pg/mL, reference range 4.2–48.8; and 127 nmol/L, reference range 60–353, respectively).To rule out a partial adrenal insufficiency (AI), a low dose short Synacthen test (LDSST) was performed using 1 µg of synthetic ACTH, with serial cortisol measurements at 0, 30, and 60 minutes. It showed cortisol peak of 407 nmol/L at 30 minutes, lower than expected (values <440 best predict AI, while values >600 nmol/L best rule out AI) (*A*, Adrenal hypoplasia) (20). Despite a borderline peak cortisol response at LDSST, the threefold increment from baseline and the absolute rise >190 nmol/L suggested preserved adrenal reserve (21). Given the absence of clear evidence of adrenal insufficiency, long-term replacement therapy was not initiated. Instead, hydrocortisone therapy was recommended during illness, injury, or prior to any surgical procedure, at a dosage of 10 mg/m^2^. To guarantee safety and monitor for progressive gland exhaustion, the adrenal stress response was re-evaluated annually throughout the follow-up period, confirming a stable borderline adrenal reserve.

The severe growth failure of our patient prompted a comprehensive evaluation of the entire pituitary axis. There was no history of familial short stature, as the mother’s height was 165.6 cm (0.51 SDS) and the father’s height was 178 cm (0.24 SDS). The patient’s mid-parental target height was calculated as 177.8 cm (−0.21 SDS), indicating a marked discrepancy with his severe growth failure, as illustrated in the growth chart (**Figure 1**). Growth hormone stimulation testing was therefore performed.

The first provocative test was performed with 25% arginine administered intravenously at a dose of 0.5 g/kg (maximum 30 g) over 30 minutes, with serial GH sampling at −30, 0, 30, 60, 90, and 120 minutes. The second provocative test was an insulin tolerance test (ITT) performed with regular insulin at a dose of 0.1 U/kg, with serial measurements of insulin, glucose, and cortisol at 0, 15, 30, 45, 60, 90, and 120 minutes. Serum GH was measured using a chemiluminescent immunoassay on the IMMULITE 2000 platform (Siemens Healthineers), calibrated against the WHO International Standard 98/574, and a diagnostic cut-off of <8 ng/mL was applied in accordance with Italian regulations.

At the provocative tests with arginine and insulin, the GH peak levels were respectively 4.6 ng/mL and 7.9 ng/mL at 30 minutes (normal values >8 ng/mL), revealing a GHD. Consistent with GHD, serum insulin-like growth factor 1 (IGF-1) levels were low for his age (48.2 ng/mL, -2.13 SD). The bone age was equal to chronological age. A brain magnetic resonance (MRI) showed a normal pituitary gland, normal posterior pituitary position, and normal hypothalamic region anatomy.

To study all the pituitary function, thyroid function was investigated, resulting in normal range: FT4 8.1 pg/mL (normal values 6.1-10.6), TSH 1.37 μUI/mL (normal values 0.79-5.85). Basal glucose metabolism was in the normal range (fasting glucose 93 mg/dL, fasting insulin 2.1 μUI/ml, HbA1c 5.5%/37 mmol/mol), while oral glucose tolerance test (OGTT) showed impaired glucose tolerance (IGT) (glycemia after 120 minutes: 141 mg/dL).

The long-term therapeutic goals of rhGH treatment were to improve linear growth, promote catch-up growth toward the patient’s genetic target height, optimize body composition and metabolic health, and prevent the adverse consequences of persistent growth failure. Treatment was planned to continue until attainment of near-adult height or growth completion, provided that efficacy and safety were maintained throughout follow-up. The parents were comprehensively informed about the risk-benefit balance of the treatment, including the theoretical potential oncological risks associated with rhGH therapy in the context of SAMD9 mutations. Following this thorough discussion, they provided fully aware informed consent, actively participating in all scheduled follow-up visits and monitoring protocols. He started recombinant human growth hormone (rhGH) therapy at a dose of 23 µg/kg/day. This initial dosage was intentionally selected at the lower end of the standard pediatric range (25–35 µg/kg/day) as a conservative, precautionary approach, specifically tailored to minimize any theoretical oncological or proliferative risk in the context of the patient’s underlying SAMD9 mutation.

During follow-up, a rigorous multidisciplinary surveillance protocol was implemented. Clinical evaluations, growth velocity assessment and safety laboratory tests were scheduled on a semi-annual basis. These included serum electrolytes, complete hepato-renal function, albumin, lactate dehydrogenase, lipid profile, inflammatory markers and venous blood gas analysis, as well as complete blood count, immunoglobulins, haptoglobin and lymphocyte subpopulations (including B-lymphocyte subsets and recent thymic emigrants). A comprehensive endocrine profile (thyroid function, gonadal axis and prolactin) was regularly assessed. Importantly, a LDSST was performed annually to monitor adrenal function over time. IGF-1 levels were systematically monitored during rhGH therapy and dynamically interpreted according to sex- and bone-age–adjusted reference ranges, with rhGH dose titrated to maintain IGF-1 within the normal range and consistently below +2 SDS.

The patient’s response to rhGH has been remarkable. After 1 year of treatment, height was 117.5 cm (-1.70 SDS), with an increase (Δ height) of +1.06 SDS, normal IGF-1 levels (93.6 ng/mL, -0.76 SD) and no side effects. After almost six years of rhGH treatment (last dose 35 µg/kg/day), at the age of 11 years and 11 months, he was still prepubertal (Tanner stage G1, Ph1, testicular volume 2 mL), his height was 135.9 cm (-1.87 SDS), with a growth velocity of 4.59 cm/year (-0.39 SDS), BMI 15.92 kg/m^2^ (-1.37 SDS), and IGF-1 levels were in the normal range (149 ng/ml, -1.66 SDS). This represents a marked catch-up growth in a child with profound pre-treatment growth failure (**Figure 1**).

During follow-up, at the age of 9.5 years, the scrotum was found uninhabited. The assessment of hypothalamic-pituitary-gonadal function showed normal values for age: LH 0.2 mUI/mL (normal values 0.2-1.7), FSH 0.60 mUI/mL (normal values 0.23-2.32), testosterone 0.1 ng/mL (normal values 0.1-0.2), AMH 39.70 ng/mL (normal values 11.8-155.34). At the ultrasound scan, the right testis was visible at the mid to distal third of the inguinal canal, the left was in the abdominal wall, at the entrance to the internal inguinal ring, both very small in size (10 mm on the right and 14 mm on the left), structurally quite homogeneous. Orchiopexy was performed, and the testes were much smaller than the norm for age with apparent complete dissociation of the epididymis.

At the most recent follow-up, at the age of 12 years, the LDST revealed adrenal function at the lower limit of normal, with a peak cortisol level of 427 nmol/L (<550 nmol/L) and an absolute increment from baseline of 200 nmol/L (basal cortisol was 227 nmol/L). While no episodes of adrenal crisis have been reported to date, a precautionary recommendation for stress-dose hydrocortisone therapy was issued for use during prolonged infections. Regarding glucose metabolism, fasting blood glucose at the last follow-up was 91 mg/dL.

In view of the impaired glucose tolerance at baseline and the known association between rhGH therapy and insulin resistance, glucose metabolism was closely monitored throughout follow-up by regular assessments of fasting plasma glucose, insulin and glycated hemoglobin (HbA1c). At the last follow-up, fasting glucose was 88 mg/dL, fasting insulin 2.9 μU/mL and HbA1c 5.9% (41 mmol/mol), indicating preserved metabolic control without progression to overt diabetes.

## Discussion

We reported the first documented case of confirmed GHD in a boy with MIRAGE syndrome who was treated with rhGH. The patient demonstrated a favorable response to six years of treatment, achieving an increase in height of nearly +1 standard deviation score (SDS) since the start of therapy. Furthermore, at the last follow-up, his growth velocity had improved from -4.56 SDS to -0.73 SDS over the previous two years (4.58 cm/year, -0.73 SDS), with no observed side effects.

Several syndromes include endocrine features, and pediatric endocrinologists contribute to developing comprehensive care strategies for patients diagnosed with specific syndromes (4, 22). The range of endocrinopathies in MIRAGE syndrome includes PAI, gonadal dysgenesis (including hypospadias and cryptorchidism), growth restriction with short stature, hypothyroidism, dysglycemia (both hyper- and hypoglycemia), and panhypopituitarism (4). Primary AI was the reason for the identification in the first two reports and seemed to be a relatively consistent feature, in approximately 70-80% of individuals with pathogenic *SAMD9* variants (1–3). They usually present with skin hyperpigmentation at birth and may develop severe dehydration and hypotension, which can be life-threatening. The diagnosis of PAI is usually confirmed by low basal or stimulated cortisol concentrations with elevated ACTH levels, often in association with aplastic or hypoplastic adrenal glands on imaging. AI may emerge later in childhood; therefore, periodic screening for PAI is recommended in these patients (3, 4).

Nevertheless, a review by Suntharalingham et al. collecting 52 patients with MIRAGE syndrome showed that this condition is more phenotypically diverse than originally recognized: absence of the adrenal phenotype was reported in 21% of the cases, while other endocrinopathies, possibly overlooked in the past, are emerging (4). Importantly, patients without adrenal insufficiency were found to have better birth outcomes, with significantly higher birth weights (median 1515 g versus 1020 g; P < 0.05) and a significantly later gestational age at birth (median 34.5 weeks versus 31.0; P < 0.05) compared to those with PAI (4). This phenotypic variability underscores the importance of considering MIRAGE syndrome even in the absence of classic adrenal features.

Growth restriction is considered a classic feature of MIRAGE syndrome. However, these patients do not usually undergo formal stimulation testing to detect GHD and are rarely treated with rhGH. It is unknown how extensively children with MIRAGE syndrome are systematically investigated for growth failure; moreover, some may have died before significant postnatal growth impairment became evident.

Recent comprehensive data from a cohort of 243 published patients with *SAMD9/SAMD9L* syndromes confirms that growth impairment is typical in individuals with *SAMD9* mutations, although the extent of endocrine evaluation in these patients remains limited (23).

The use of rhGH therapy has so far been reported in only one MIRAGE syndrome case, without documented GHD. Shima et al. described a girl, aged 10 years 9 months, with a 46,XX karyotype, with growth retardation attributed to supraphysiological steroid dosages. She started rhGH treatment (35 mcg/kg/day) with the indication of short stature in small for gestational age (SGA), and she showed catch-up growth. Unfortunately, the patient died 1 year later from multiorgan failure due to severe infection (10). In their review, Suntharalingham et al. reported a male with panhypopituitarism and two patients with GHD (one male and one female), although no further information is available (i.e., age at diagnosis, performed stimulation tests, GH peak, IGF-1 levels, rhGH treatment, other pituitary hormones involved) (4). This paucity of data on GH evaluation and treatment in MIRAGE syndrome contrasts with the well-documented endocrine abnormalities in the condition and suggests that systematic endocrine assessment, including GH testing, may be underutilized in this population (10).

Interestingly, our patient has not developed PAI so far at the age of 11 years, consistent with reports that adrenal involvement is not universal in MIRAGE syndrome. A female Japanese patient carrying the same *SAMD9* mutation has previously been described, with severe short stature (-4.4 SDS) at the age of 6 (although GH secretion was not reported), undetectable ovaries on abdominal ultrasound, but normal adrenal function and no evidence of monosomy 7 or myelodysplastic syndrome (13).

Regarding the safety of rhGH therapy in our patient, several considerations are relevant. While MIRAGE syndrome is associated with bone marrow failure and predisposition to myelodysplasia, in the general pediatric population, current evidence suggests that GH therapy does not substantially increase the risk of leukemia in patients without pre-existing risk factors (24). However, patients with SAMD9 mutations represent a unique population with inherent risk for developing monosomy 7 and MDS through somatic genetic rescue mechanisms. Approximately two-thirds of patients with *SAMD9/SAMD9L* syndromes undergo somatic genetic rescue, which can involve monosomy 7 that may spontaneously resolve (transient monosomy 7) or progress to MDS/leukemia (7, 23). Importantly, rhGH therapy in this context represents a physiological replacement aimed at restoring normal hormonal balance and mitigating GHD-associated adverse effects on body composition, bone health, and metabolic outcomes. Given this context, our patient’s favorable 6-year outcome without hematologic complications is reassuring, though continued surveillance remains essential (23, 24).

The mechanism underlying GHD in MIRAGE syndrome remains speculative. SAMD9 gain-of-function mutations are known to exert broad antiproliferative effects and to perturb cellular signaling, and it is conceivable that the pituitary gland may be particularly vulnerable to these effects, potentially explaining the occurrence of panhypopituitarism and isolated GHD in some patients (1).

To better define a potential causal link between impaired GH secretion and short stature in MIRAGE syndrome, GH secretion and the efficacy of GH replacement therapy should be evaluated in a larger number of affected individuals. Rather than routine testing in all cases, systematic assessment of GH secretion with stimulation tests should be considered in children with MIRAGE syndrome who present with significant growth failure or other suggestive features of pituitary dysfunction. In patients with confirmed GHD, prompt initiation of recombinant human GH (rhGH) replacement therapy may significantly improve their growth trajectory, while decisions should be individualized and made in close collaboration with hematology/oncology teams.

Furthermore, rhGH therapy effectively addresses the significant psychosocial burden and emotional or behavioral adjustment difficulties often associated with short stature in pediatric patients compared to their normal-statured peers, thereby profoundly improving their long-term quality of life (24).

The clinical course and management of our patient align with the recent shifting paradigm in MIRAGE syndrome literature. While early reports focused predominantly on the severe, life-threatening infantile complications, recent evidence highlights a growing cohort of patients surviving into adolescence and adulthood. In a comprehensive review by Yamada et al. (25) focusing on long-term survival in MIRAGE syndrome, the authors elucidated that surviving patients exhibit a more nuanced spectrum of chronic systemic manifestations, emphasizing that long-term multi-organ endocrine management becomes a cornerstone of care as these patients age (25).

Given the complex multisystem nature of MIRAGE syndrome and the potential for progressive endocrinopathies, a comprehensive endocrine evaluation, including assessment of the entire hypothalamic–pituitary axis, should be undertaken in patients with SAMD9 mutations. Such systematic evaluation would help clarify the true prevalence of GHD and other pituitary defects in this population and support the development of evidence-based recommendations for rhGH therapy, while ensuring ongoing hematologic surveillance in view of the inherent risk of bone marrow failure and clonal evolution associated with these syndromes (26).

## Conclusion

This case illustrates that rhGH therapy can be effective and well tolerated in a child with MIRAGE syndrome and GHD, leading to substantial catch-up growth without apparent medium-term hematologic complications over a six year period. Our observations highlight the importance of structured endocrine assessment, including GH stimulation testing, in MIRAGE syndrome patients with marked growth failure, and suggest that rhGH replacement may be a valuable therapeutic option when GHD is documented. Larger, systematically characterized cohorts with long-term follow-up are needed to define the prevalence of GHD in MIRAGE syndrome, to better delineate the risk–benefit profile of rhGH treatment, and to inform future clinical guidelines in this high-risk population.

## Data Availability

The raw data supporting the conclusions of this article will be made available by the authors, without undue reservation.
